# On the Computational Study of a Fully Wetted Longitudinal Porous Heat Exchanger Using a Machine Learning Approach

**DOI:** 10.3390/e24091280

**Published:** 2022-09-11

**Authors:** Hosam Alhakami, Naveed Ahmad Khan, Muhammad Sulaiman, Wajdi Alhakami, Abdullah Baz

**Affiliations:** 1 Department of Computer Science, College of Computer and Information Systems, Umm Al-Qura University, Makkah 21955, Saudi Arabia; 2 Department of Mathematics, Abdul Wali Khan University, Mardan 23200, Pakistan; 3 Department of Information Technology, College of Computers and Information Technology, Taif University, Taif 26571, Saudi Arabia; 4 Department of Computer Engineering, College of Computer and Information Systems, Umm Al-Qura University, Makkah 21955, Saudi Arabia

**Keywords:** wet porous fin, functionally graded materials, thermal analysis, meta-heuristics, machine learning techniques

## Abstract

The present study concerns the modeling of the thermal behavior of a porous longitudinal fin under fully wetted conditions with linear, quadratic, and exponential thermal conductivities surrounded by environments that are convective, conductive, and radiative. Porous fins are widely used in various engineering and everyday life applications. The Darcy model was used to formulate the governing non-linear singular differential equation for the heat transfer phenomenon in the fin. The universal approximation power of multilayer perceptron artificial neural networks (ANN) was applied to establish a model of approximate solutions for the singular non-linear boundary value problem. The optimization strategy of a sports-inspired meta-heuristic paradigm, the Tiki-Taka algorithm (TTA) with sequential quadratic programming (SQP), was utilized to determine the thermal performance and the effective use of fins for diverse values of physical parameters, such as parameter for the moist porous medium, dimensionless ambient temperature, radiation coefficient, power index, in-homogeneity index, convection coefficient, and dimensionless temperature. The results of the designed ANN-TTA-SQP algorithm were validated by comparison with state-of-the-art techniques, including the whale optimization algorithm (WOA), cuckoo search algorithm (CSA), grey wolf optimization (GWO) algorithm, particle swarm optimization (PSO) algorithm, and machine learning algorithms. The percentage of absolute errors and the mean square error in the solutions of the proposed technique were found to lie between $10^{-4}$ to $10^{-5}$ and $10^{-8}$ to $10^{-10}$, respectively. A comprehensive study of graphs, statistics of the solutions, and errors demonstrated that the proposed scheme’s results were accurate, stable, and reliable. It was concluded that the pace at which heat is transferred from the surface of the fin to the surrounding environment increases in proportion to the degree to which the wet porosity parameter is increased. At the same time, inverse behavior was observed for increase in the power index. The results obtained may support the structural design of thermally effective cooling methods for various electronic consumer devices.

## 1. Introduction

The problem of efficient cooling in electronic systems has attracted much attention for many reasons. These include the trend toward downsizing in electronic packaging design which requires a more compact volume with higher performance standards [1]. Improving overall effectiveness frequently requires increasing both power and on-chip power density. This is connected to an increase in heat that must be dissipated, so raising either of these factors might be problematic. Consequently, the efficient removal of heat produced by modern electronic systems has emerged as a critical issue for the design processes used by both electrical and mechanical engineers [2]. Fins are extended surfaces that have been developed either to improve the heat transfer rate while maintaining a constant surface temperature or to reduce the surface temperature while maintaining a constant heat transfer rate. Fins can be found on radiators, heat exchangers, and other devices, and are a concept studied in the field of heat transfer [3]. The amount of heat present can be used as a basis for calculating the quantity of heat an item can transmit by conduction, convection, or radiation. Increasing heat transmission may be accomplished in one of three ways: by boosting the temperature difference between an object and its surroundings, expanding the coefficient of the convective heat transfer coefficient, or enhancing the object/fin surface area [4,5]. Pursuing either of the first two alternatives is not feasible or cost-effective in some situations. Adding a fin causes an increase in surface area. Therefore, doing so can occasionally be an economical solution to problems associated with the transfer of heat [6,7].

Many practical applications in various industrial, electrical, and mechanical engineering domains, such as gas turbines, bike heads, aircraft engines, automobiles, and heat sinks utilize fin structures to provide increased surface area and, as a result, to enhance the efficiency of heat transfer. Kiwan and Nimr [8] modeled the performance of a permeable fin, and considered the functioning of porous fins compared to solid fins. They found that the thermodynamic efficiency of porous heat exchangers was much higher than that of solid heat exchangers of identical weight [9]. Kiwan [10] presented a simplified model for investigating how well a porous fin performs in an environment dominated by natural convection under a variety of tip conditions. The author utilized Darcy’s model and energy balance method to compile the flow and geometric characteristics into a dimensionless number called $S_{H}$, and studied this number’s influence on the heat transfer rate. Gawai and Mathew [11] proposed a heat enhancement approach in which depressions on the surface of aluminum and brass were used in place of projections to achieve the desired effect. These caused a phenomenon known as “scrubbing of the fluid” which speeds up the heat transfer process by reducing pressure loss. Shouman [12] conducted an extensive study on the impact of internal heat production/generation, thermal conductivity (temperature-dependent), and magnetic flux on the transfer of heat through a porous heat exchanger with single phase fluid flow.

Heat transfer rates are improved by using porous extended surfaces which often outperform traditional solid fins in many applications [13]. In the past, many investigations have been undertaken on porous fins. Kiwan introduced a numerical approximating approach, the finite volume method (FVM), while Zeitoun [14] predicted the thermic performance of a porous heat exchanger that was affixed to the inner layer of the annular gap created between two concentric cylindrical enclosures. It was found that the porous fin enhanced the rate of heat transmission in comparison to the traditional solid fin. Sharqawy [15] investigated the effectiveness of straight fins in various configurations when simultaneously subjected to various processes of heat and mass transfer. Domairry [16] utilized the homotopy analysis method (HAM) to simplify a non-linear governing mathematical model pertaining to the temperature distribution in a straight fin.

Generally, singular non-linear differential equations are used to model the behavior of porous heat exchangers in radiative-convective-conductive environments with internally generated heat and heat conductance (temperature-dependent). The non-linear fin problem has received considerable attention because of its industrial applications. As a result, a wide variety of numerical and analytical approaches have been established to solve fin equations for the approximate solutions [17]. Chiu [18] implemented the Adomian decomposition technique (ADM) to make an approximation of the ideal/efficient geometrical shape of a longitudinal fin subjected to convective surroundings or environments with heat (thermal) conductance. Chang [19] provided an approximation of an analytical solution for heat transfer models in multiple environments. Hatami [20] investigated the heat and thermic distribution for a porous longitudinal radiative-convective heat exchanger of $Si_{3}N_{4}$ material with four different shapes (exponential, convex, rectangular, and triangular) using a statistical approach to calculate the optimal fitting of solutions with a least square method (LSM). Later, Moitsheki [21] extended the perturbation method to simulate analytical solutions for non-linear problems, describing the thermic distribution of heat in a one-dimensional longitudinal radial heat exchanger for concave, rectangular, triangular and convex parabolic profiles. In [22], the Adomian decomposition technique was utilised to determine the optimal design parameters pertinent to a moving porous extended surface. It was concluded that the extended surface of exponential designs was highly efficient. A finite-difference method was adopted by Sobamowo [23] to study the total heat transfer, fin efficiency, and internal heat generation of a fin with thermo-geometric properties. A two-dimensional differential transform scheme was applied by P. L. Ndlovu [24] to study transient heat transfer of different configurations (convex parabolic and concave parabolic) of longitudinal extended surfaces. N. A. Khan [25,26] modeled approximate solutions using computer-assisted, global and local optimization techniques for the governing non-linear model of convective-conductive-radiative heat exchangers with conductance to heat. M. Nabati [27] studied the effect of various physical parameters on porous fins by discretizing the governing non-linear differential equation using the Sinc collocation method (SCM). In [28], the partial Noether method (PNM) was employed to explore the impacts of the electromagnetic flux, radiation coefficient, thermo-geometric parameter, and non-linear conductance on the thermic behavior of a radiative-convective straight fin.

Some other numerical techniques applied to the solution of fin design problems include the Akbari–Ganji method (AGM) [29], the Haar wavelet quasilinearization method [30], the Legendre wavelet collocation method (LWCM) [31], the Hermite wavelet method (HWM) [32], and the integral transform method (ITM) [33]. These techniques have been applied to solve non-linear, steady, and unsteady problems. As well as their advantages, they have some limitations. The major drawback of these approaches is that they can only be applied to certain subsets of a relatively limited class of mathematical problems. Most of these techniques are iterative and gradient-based procedures requiring prior information about the problem. Prior information includes smoothness, continuity, differentiability, gradient, choice of initial guess, and smallness of parameters. It is of note that such techniques are gradient-based and require information about the problem beforehand. The availability of several local optima, which leads to solutions where global optimality cannot be easily ensured, is one of the fundamental limitations of such gradient-based approaches. Global optimality is sought in gradient-based approaches by randomly scanning the design space from various starting points. However, this causes the technique to become sluggish and computationally inefficient for complex non-linear optimization problems. On the other hand, metaheuristic (MH) optimization algorithms have been quite popular in recent years due to their numerous benefits over traditional numerical schemes [34]. In general, the impetus for the use of MH algorithms comes from a wide variety of chemical and physical phenomena. These algorithms are designed to imitate a wide range of physical and/or chemical events, such as movements, electrical charges, gravitational forces, hunting strategies, and so on. Approaches based on MH can be used to solve problems that have many objectives, a wide variety of solutions, and non-linear formulations. As a result, they are used to develop high-quality approximations and solutions to an ever-increasing variety of intricate problems that arise in the real world.

In this paper, numerical solutions for fully wetted longitudinal porous heat exchangers with different thermal conductivities are calculated based on the simple concept of artificial intelligence (AI), implemented through the application of neural networks and optimization procedures of meta-heuristic techniques [35,36,37,38,39]. Recently, artificial intelligence-based stochastic techniques have been successfully implemented for various problems in different domains of reaction kinematics [40,41], marine engineering [42], wireless communication [43], and fluid dynamics [44,45,46]. These applications motivated the authors to design a novel unsupervised technique using the computational approximation ability of layer structure feed-forward ANNs, the global and local optimization of the Tiki-Taka algorithm (TTA), and sequential quadratic programming (SQP). The designed ANN-TTA-SQP algorithm was applied to different problems relating to fully wetted longitudinal porous heat exchangers of multiple conductance. The designed ANN-TTA-SQP algorithm was consistently found to be correct compared to the results of the whale optimization algorithm (WOA), the cuckoo search algorithm (CSA), the grey wolf optimization (GWO) algorithm, the particle swarm optimization (PSO) algorithm, and machine learning algorithms. Apart from the efficiency and accuracy of solutions obtained with neural network methodologies, other advantages of the proposed technique, in comparison to traditional numerical methods and classical numerical methods, are as follows:

- The MH approach developed does not make use of gradients and does not call on any previous knowledge (e.g., initial guess, initial approximation, continuity, differentiability and small auxiliary parameters) of the problem. Unlike other deterministic approaches, the ANN-TTA-SQP only requires initial parameter settings (e.g., max. iterations, population size, etc.) and execution stopping criteria.
- A simple method is provided that enables the singularity and non-linearity of complex systems, such as longitudinal porous heat exchangers, to be successfully dealt with.
- Stochastic approaches based on ANN, in contrast to deterministic solvers, are capable of providing a continuous solution across the entirety of the integration domain.

The stability, efficiency and precision of the newly proposed scheme were assessed through performance indicators, including the mean error in Nash–Sutcliffe efficiency (ENSE), absolute deviations, root mean square error (RMSE), and Theil’s inequality coefficient (TIC).

## 2. Mathematical Formulation of Physical Problem

**Figure 1:**
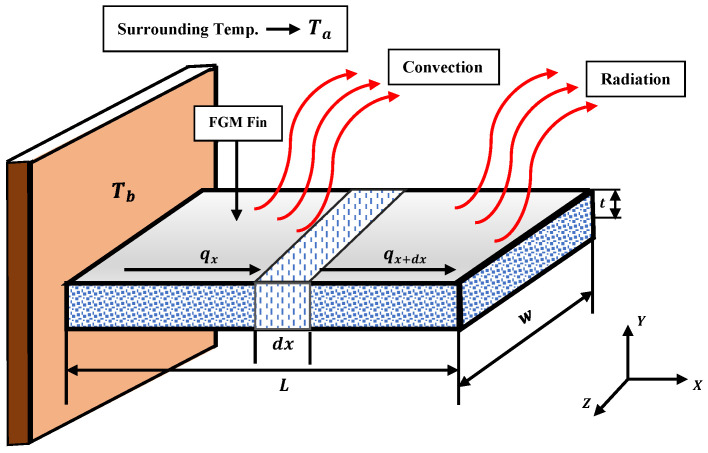
A diagrammatic representation of the porous longitudinal fin model, illustrating natural phenomena of radiation and convection.

The physical problems associated with a longitudinal fin with natural convection and radiation of length (L), width (w), and cross-sectional area (A) placed on a surface maintained at a temperature $T_{b}$ are represented in Figure 1. The entire matrix of the solid fin is saturated (wetted), and single-phase fluid is assumed to fill the fin medium, which is isotropic, saturated, and homogenous. The Darcy model was utilised to investigate the process of movement of fluid via pores. The steady-state energy balance equation around the small cross-sectional area (dx) of the fin is given

(1) $$q\left(x\right)+\dot{m}c_{p}\left(T_{a}-T\right)-q\left(x+dx\right)=whdx\left(\phi -1\right)\left(T_{a}-T\right)-\varepsilon w\sigma dx\left(T_{a}^{4}-T^{4}\right)+h_{D}wdxi_{fg}\left(\phi -1\right)\left(\omega_{a}-\omega \right),$$

where, *h* represents the coefficient of transmission in heat, *q* is the heat transfer rate of base, ε is the surface emissivity of fin, $c_{p}$ is specific heat, ϕ is the porosity parameter, ω is the saturation level of humidity in the air, $i_{fg}$ is the heat that is released from water during evaporation, σ is the Stefan–Boltzmann constant, $\omega_{a}$ represents the humidity ratio of the surrounding air and $\dot{m}$ is the mass flow rate of the fluid which is defined as [47]

(2) $$\dot{m}=\rho_{f}v\left(x\right)wdx,$$

where, $rho_{f}$ is the density of the fluid being measured, and the velocity of fluid along the axial direction is v(x). Darcy’s law dictates that it should be given as

(3) $$v\left(x\right)=-gK\beta_{f}\left(T_{a}-T\right){\left(\nu_{f}\right)}^{-1},$$

here, $\beta_{f}$, *K* and $\nu_{f}$, are the volumetric thermal expansion and the permeability and kinematic viscosity of fluid, respectively. From Fourier’s law of conduction, the heat transfer rate can be defined as

(4) $$q=-k\left(x\right)tw\frac{dT}{dx},$$

where, k(x) is the thermal conductivity, and *h* is the convective heat transfer coefficient which is given as

(5) $$h=h_{b}{\left[\frac{T-T_{a}}{T_{b}-T_{a}}\right]}^{p}=h_{D}C_{p}Le^{\frac{2}{3}},$$

where, *p* is a power index that measures the nature/strength of different fluid flows. In the case of functionally graded material (FGM), thermal conductivity is affected by the length of a fin. In this study, we focused on three different examples of the FGM, including linear, quadratic, and exponential fluctuations in heat conductivity. For each case the heat conductance is defined as

(6) $$k\left(x\right)=k_{0}\left(ax+1\right),$$

(7) $$k\left(x\right)=k_{0}\left(ax^{2}+1\right),$$

(8) $$k\left(x\right)=k_{0}e^{ax}.$$

Substituting Equations (2)–(5) with Equations (6)–(8) results in the governing differential equation models for linear, quadratic and exponential FGM as

(9) $$\begin{array}{cc} \frac{d}{dx}\left(\left(1+ax\right)\frac{dT}{dx}\right) & +\frac{\rho g\beta_{f}KC_{p}}{\nu_{f}k_{0}t}{\left(T_{a}-T\right)}^{2}+\frac{\sigma \varepsilon }{k_{0}t}\left(T_{a}^{4}-T^{4}\right)\\ \; & -\frac{h_{a}\left(\phi -1\right){\left(T_{a}-T\right)}^{1+p}}{k_{0}t{\left(T_{b}-T_{a}\right)}^{p}}-\frac{h_{D}i_{fg}\left(\phi -1\right)\left(\omega_{a}-\omega \right)}{k_{0}t}=0, \end{array}$$

(10) $$\begin{array}{cc} \frac{d}{dx}\left(\left(1+ax^{2}\right)\frac{dT}{dx}\right) & +\frac{\rho g\beta_{f}KC_{p}}{\nu_{f}k_{0}t}{\left(T_{a}-T\right)}^{2}+\frac{\sigma \varepsilon }{k_{0}t}\left(T_{a}^{4}-T^{4}\right)\\ \; & -\frac{h_{a}\left(\phi -1\right){\left(T_{a}-T\right)}^{1+p}}{k_{0}t{\left(T_{b}-T_{a}\right)}^{p}}-\frac{h_{D}i_{fg}\left(\phi -1\right)\left(\omega_{a}-\omega \right)}{k_{0}t}=0, \end{array}$$

(11) $$\begin{array}{cc} \frac{d}{dx}\left(\left(e^{ax}\right)\frac{dT}{dx}\right) & +\frac{\rho g\beta_{f}KC_{p}}{\nu_{f}k_{0}t}{\left(T_{a}-T\right)}^{2}+\frac{\sigma \varepsilon }{k_{0}t}\left(T_{a}^{4}-T^{4}\right)\\ \; & -\frac{h_{a}\left(\phi -1\right){\left(T_{a}-T\right)}^{1+p}}{k_{0}t{\left(T_{b}-T_{a}\right)}^{p}}-\frac{h_{D}i_{fg}\left(\phi -1\right)\left(\omega_{a}-\omega \right)}{k_{0}t}=0. \end{array}$$

The rate of heat transmission is practically negligible at the fin tip because the thickness of the fin is less. As a result, the following boundary criteria must be satisfied for the adiabatic tip fin:

(12) $$\begin{array}{cc} \; & \;\mathrm{at}\;x=0\;T\left(0\right)=T_{b},\\ \; & \;\mathrm{at}\;x=L\;\frac{dT(L)}{dx}=0. \end{array}$$

The following dimensionless parameters are employed to reduce Equations (9)–(11) to a dimensionless form.

(13) $$\begin{array}{c} \theta =\frac{T}{T_{b}},\;Nc=\frac{\rho g\beta_{f}KC_{p}T_{b}L^{2}}{\nu_{f}k_{0}t},\;\theta_{a}=\frac{T_{a}}{T_{b}},\;X=\frac{x}{L},Nr=\frac{\varepsilon \sigma L^{2}T_{b}^{3}}{k_{0}t},\\ m_{0}=\frac{h_{b}L^{2}\left(1-\phi \right)}{k_{0}t},\;m_{1}=-\frac{h_{b}i_{fg}b_{2}\left(\phi -1\right)L^{2}}{k_{0}tC_{p}Le^{2}},\;\left(\omega_{a}-\omega \right)=b_{2}\left(T_{a}-T\right). \end{array}$$

Linear FGM:

(14) $$\frac{d^{2}\theta }{dX^{2}}+X\beta \frac{d^{2}\theta }{dX^{2}}+\beta \frac{d\theta }{dX}+Nr\left(\theta_{a}^{4}-\theta^{4}\right)+Nc{\left(\theta_{a}-\theta \right)}^{2}-\frac{m_{2}{\left(\theta_{a}-\theta \right)}^{1+p}}{{\left(\theta_{a}-1\right)}^{p}}=0,$$

Quadratic FGM:

(15) $$\frac{d^{2}\theta }{dX^{2}}+\alpha X^{2}\frac{d^{2}\theta }{dX^{2}}+2\alpha X\frac{d\theta }{dX}+Nr\left(\theta_{a}^{4}-\theta^{4}\right)+Nc{\left(\theta_{a}-\theta \right)}^{2}-\frac{m_{2}{\left(\theta_{a}-\theta \right)}^{1+p}}{{\left(\theta_{a}-1\right)}^{p}}=0,$$

Exponential FGM:

(16) $$e^{\beta X}\frac{d^{2}\theta }{dX^{2}}+\beta e^{\beta X}\frac{d\theta }{dX}+Nr\left(\theta_{a}^{4}-\theta^{4}\right)+Nc{\left(\theta_{a}-\theta \right)}^{2}-\frac{m_{2}{\left(\theta_{a}-\theta \right)}^{1+p}}{{\left(\theta_{a}-1\right)}^{p}}=0,$$

where, Nr, $m_{2}$, $N_{c}$, $\theta_{a}$, *X*, and θ, are the radiation coefficient, wet porous parameter for a moist porous medium, convection coefficient, non-dimensional ambient temperature, dimensionless length and temperature, respectively.

In dimensionless terms, the boundary conditions for Equations (14)–(16) are outlined below:

(17) $$\begin{array}{c} \theta =1\;\;\mathrm{at}\;X=0,\\ \theta^{\prime }=0\;\;\mathrm{at}\;X=1. \end{array}$$

## 3. Proposed Methodology

The framework for the proposed meta-heuristic technique is divided into two stages. Initially, an unsupervised objective function for the models in Equations (14)–(16) of fully wetted longitudinal porous heat exchangers for linear, quadratic and exponential cases is constructed with ANN modeling. Then the objective function is minimized by training the weights or neurons in the ANN architecture using sports inspired by the Tiki-Taka algorithm for global exploration and sequential quadratic programming for local exploitation.

### 3.1. Neural Networks Based Differential Equation Models

In recent years, there has been a significant increase in the reporting of the application of ANNs to the solution of differential equations that include both integer and fractional derivatives. The mathematical model for the approximate numerical solution of Equations (14)–(16) is formulated in the following form

(18) $$\hat{\theta }=\sum_{i=1}^{H}\tilde{\xi_{i}}f(\tilde{a_{i}}X+\tilde{b_{i}}),$$

where, $\hat{\theta }$ is the neural network output (approximate solution) with input vector *X*. $\tilde{\xi_{i}}=\left[{\tilde{\xi }}_{1},{\tilde{\xi }}_{2},{\tilde{\xi }}_{3},\ldots ,{\tilde{\xi }}_{H}\right]$, $\tilde{a}=\left[{\tilde{a}}_{1},{\tilde{a}}_{2},{\tilde{a}}_{3},\ldots ,{\tilde{a}}_{h}\right]$ and $\tilde{b_{i}}=\left[{\tilde{b}}_{1},{\tilde{b}}_{2},{\tilde{b}}_{3},\ldots ,{\tilde{b}}_{H}\right]$ represents the corresponding vector of adjustable weight parameters, and *m* is the number of neurons. *f* is the Log-sigmoid activation function and has the form $\frac{1}{1+e^{-x}}$.

Now, the first and second derivative of the network output is given as

(19) $$\frac{d\hat{\theta }}{dX}=\sum_{i=1}^{H}\tilde{\xi_{i}}\frac{d}{dX}f\left(\tilde{a_{i}}X+\tilde{b_{i}}\right)=\sum_{i=1}^{k}\tilde{\xi_{i}}\tilde{a_{i}}\frac{e^{-\left(\tilde{a_{i}}X+\tilde{b_{i}}\right)}}{1+e^{-{\left(\tilde{\xi_{i}}X+\tilde{b_{i}}\right)}^{2}}},$$

(20) $$\frac{d^{2}\hat{\theta }}{dX^{2}}=\sum_{i=1}^{H}\tilde{\xi_{i}}\frac{d^{2}}{dX^{2}}f\left(\tilde{a_{i}}X+\tilde{b_{i}}\right)=\sum_{i=1}^{k}\tilde{\xi_{i}}{\tilde{a_{i}}}^{2}\left(\frac{2e^{-2\left(\tilde{a_{i}}X+\tilde{b_{i}}\right)}}{{\left(1+e^{-\left(\tilde{a_{i}}X+\tilde{b_{i}}\right)}\right)}^{3}}-\frac{e^{-\left(\tilde{a_{i}}X+\tilde{b_{i}}\right)}}{{\left(1+e^{-\left(\tilde{a_{i}}X+\tilde{b_{i}}\right)}\right)}^{2}}\right).$$

A combination of Equations (18)–(20) is used to construct a fitness function of the problem in Equations (14)–(16) using the error in an unsupervised manner (based on the sum of mean-squared errors) which is defined as follows

(21) $$Minimize\;ℑ={ℑ}_{1}+{ℑ}_{2},$$

where, ${ℑ}_{1}$ and ${ℑ}_{2}$ correspond to the mean square error functions of the differential equation and the boundary conditions, respectively. For linear, quadratic and exponential FGM, ${ℑ}_{1}$ is defined as

(22) $${ℑ}_{1}=\frac{1}{N}\sum_{k=1}^{N}{\left(\frac{d^{2}{\hat{\theta }}_{k}}{dX^{2}}+X\beta \frac{d^{2}{\hat{\theta }}_{k}}{dX^{2}}+\beta \frac{d{\hat{\theta }}_{k}}{dX}-Nc{\left({\hat{\theta }}_{k}-\theta_{a}\right)}^{2}-Nr\left({\hat{\theta }}_{k}^{4}-\theta_{a}^{4}\right)-\frac{m_{2}{\left({\hat{\theta }}_{k}-\theta_{a}\right)}^{p+1}}{{\left(1-\theta_{a}\right)}^{p}}\right)}^{2},$$

(23) $${ℑ}_{1}=\frac{1}{N}\sum_{k=1}^{N}{\left(\frac{d^{2}{\hat{\theta }}_{k}}{dX^{2}}+\alpha X^{2}\frac{d^{2}{\hat{\theta }}_{k}}{dX^{2}}+2\alpha X\frac{d{\hat{\theta }}_{k}}{dX}-Nc{\left({\hat{\theta }}_{k}-\theta_{a}\right)}^{2}-Nr\left({\hat{\theta }}_{k}^{4}-\theta_{a}^{4}\right)-\frac{m_{2}{\left({\hat{\theta }}_{k}-\theta_{a}\right)}^{p+1}}{{\left(1-\theta_{a}\right)}^{p}}\right)}^{2},$$

(24) $${ℑ}_{1}=\frac{1}{N}\sum_{k=1}^{N}{\left(e^{\beta X}\frac{d^{2}{\hat{\theta }}_{k}}{dX^{2}}+\beta e^{\beta X}\frac{d{\hat{\theta }}_{k}}{dX}-Nr\left({\hat{\theta }}_{k}^{4}-\theta_{a}^{4}\right)-Nc{\left({\hat{\theta }}_{k}-\theta_{a}\right)}^{2}-\frac{m_{2}{\left({\hat{\theta }}_{k}-\theta_{a}\right)}^{p+1}}{{\left(1-\theta_{a}\right)}^{p}}\right)}^{2},$$

where, N=1/h denotes the number of grid points from [0,1] and *h* is a stepsize. The error function for boundary conditions is defined as

(25) $${ℑ}_{2}=\frac{1}{2}\left({\left(\hat{\theta }\left(0\right)-1\right)}^{2}+{\left(\frac{d\hat{\theta }\left(1\right)}{dX}-0\right)}^{2}\right).$$

It is evident from the above formulation and error term that the approximation $\hat{\theta }\left(X\right)$ approaches the original/exact solution θ(X) when the error terms ${ℑ}_{1}$ and ${ℑ}_{2}$ approach to zero.

### 3.2. Optimization Procedure

The optimization of Equation (21) is based on a hybrid procedure of global and local search optimization techniques, using the Tiki-Taka algorithm and sequential quadratic programming. A brief overview of the algorithm is provided below.

#### 3.2.1. Tiki-Taka Algorithm

Tiki-Taka is a style of play popularised by the Spanish national team and the football club of Barcelona (BCF). It is distinguished by constant movement of the players, short passes, and complete mastery of the ball at all times. Physical football, which emphasises physical power, man-marking and sprinting ability of the competitor, is diametrically opposed to the Tiki-Taka style of play. Tiki-Taka is a kind of soccer that requires only a limited number of competent players since emphasis is on rapid movements and precise placement of players. These players are the most important for the team, and their performance determines how quickly the game progresses.

**Figure 2:**
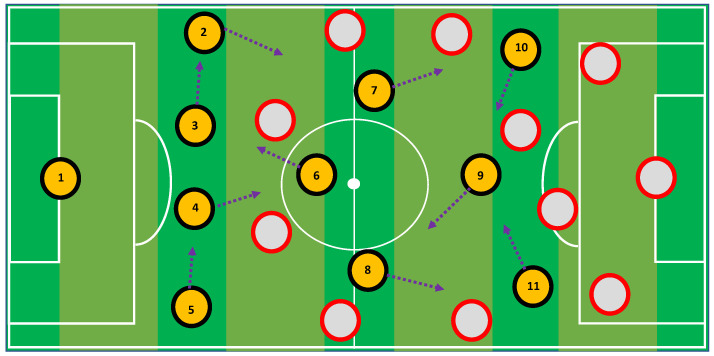
Schematic of the passing of ball between the players using Tiki-Taka style.

The Tiki-Taka Algorithm (TTA) is a meta-heuristic approach that was developed by M. Rashid [48] in 2020. It imitates the two primary features of the Tiki-Taka style, which are short rapid passing and player mobility. TTA was named after the Tiki-Taka style of play. In a real game of football, the players line up in the shape of triangles and begin tackling their opponents by passing the ball back and forth within the triangle as shown in Figure 2. The players then work to improve their position by observing where the ball and the other key players are located. The TTA models the performance of several crucial players to increase a convergence of solutions. The search strategy of TTA is divided into the following phases.

Initialization: During this phase, a football team with *n* number of players is considered using their baseline positions in *d* bounded directions. The lower and upper limits are assumed to be (LB) and (UB), respectively. Concurrently, 10% of the entire participants, or at least three individuals, are recognised as being important players, and this identification is symbolised by $n_{k}$. The matrices *B* and *P*, are used to hold information on the position/location of the ball and the players, respectively. The starting/initial positions of the players are determined by Equation (26).

(26) $$p_{i}^{t+1}=\mathrm{LB}+\mathrm{rand}\left(\right)\times \left(\mathrm{UB}-\mathrm{LB}\right).$$

The starting position of the player, is denoted by *P*, which is ranked according to the objective function. Initially, *B* is equivalent to *P*. At the end of each iteration, the placement of the key/crucial player $n_{k}$ will have been updated. Equations (27) and (28) highlight the respective matrices.

(27) $$B=\left(\begin{array}{cccc} b_{1,1} & b_{1,2} & \cdots & b_{1,d}\\ b_{2,1} & b_{2,2} & \ldots & b_{2,d}\\ \vdots & \vdots & \ddots & \vdots \\ b_{n,1} & b_{n,2} & \cdots & b_{n,d} \end{array}\right),$$

(28) $$P=\left(\begin{array}{cccc} p_{1,1} & p_{1,2} & \cdots & p_{1,d}\\ p_{2,1} & p_{2,2} & \cdots & p_{2,d}\\ \vdots & \vdots & \ddots & \vdots \\ p_{n,1} & p_{n,2} & \cdots & p_{n,d} \end{array}\right).$$

**Figure 3:**
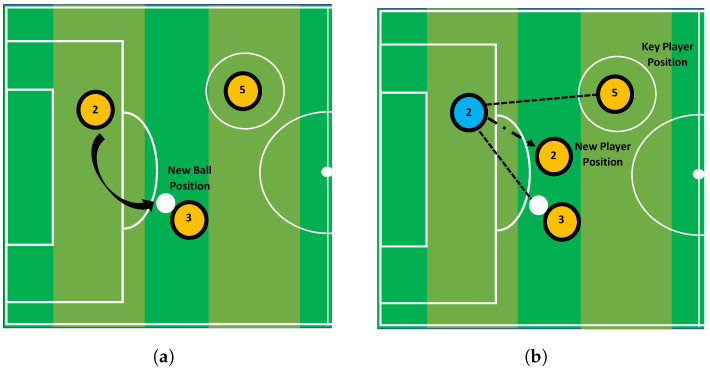
(**a**,**b**) Shows the updated positions of ball and the player during the optimization proves.

Update ball position: The TT algorithm makes use of a Tiki-Taka playing style, which places a strong emphasis on rapid passing. The ball moves from one player to the next player who is physically closest to them. Of all the passes, 10–30 percent are deemed to be failed passes. This percentage varies from 0.1 to 0.3, and is expressed using a parameter of probability *℘*. The updated position of the ball is expressed by Equation (29) and the process is shown in Figure 3.

(29) $$b_{i}^{t+1}=\left\{\begin{array}{c} \mathrm{rand}\left(b_{i}^{t}-b_{i}^{t+1}\right)+b_{i}^{t},r_{p}>℘\\ b_{i}^{t}+\left(c_{1}+\mathrm{rand}\right)\left(b_{i}^{t+1}-b_{i}^{t}\right),r_{p}\leq ℘ \end{array}\right.$$

where, $r_{p}$ is a random integer with a constant distribution. The exploitation phase (successful passes) of TTA is represented by $r_{p}>℘$ and the unsuccessful passes, i.e., the exploration phase, is signified by $r_{p}\leq ℘$. The impact of the ball’s reflection magnitude in a failed pass is represented by the coefficient $c_{1}$, which can take values between 0.5 and 1.5. The *i*th, and (i+1)th positions of the ball are denoted by $b_{i}^{t}$ and $b_{i}^{t+1}$, respectively.

Update player position: In TTA, when updating the position of the current player, the locations of both the ball and the crucial player in the action are taken into consideration, as shown in Figure 2. To determine the new position of the ith player, Equation (30) is applied.

(30) $$p_{i}^{t+1}=p_{i}^{t}+\mathrm{rand}*c_{2}*\left(b_{i}^{t}-p_{i}^{t}\right)+\mathrm{rand}*c_{3}*\left(\hbar -p_{i}^{t}\right),$$

here, *ℏ* denotes the current position of the ith player with respect to the ball and the crucial player. This is the global best position which is taken into account by the coefficients $c_{2}$ and $c_{3}$, respectively. The values of $c_{2}$ and $c_{3}$ lie between 1.0 to 2.5 and 0.5 to 1.5.

#### 3.2.2. Sequential Quadratic Programming

**Figure 4:**
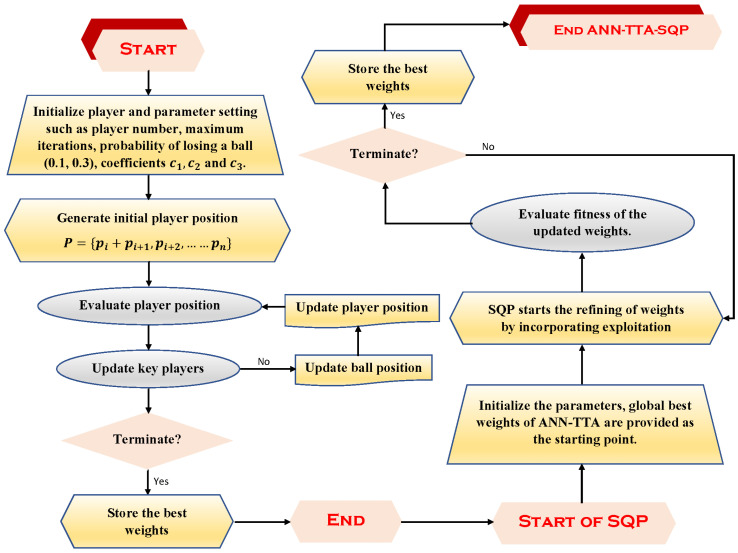
Graphical illustration of the working steps of hybrid technique of the Tiki-Taka algorithm and local search processing of SQP for the training/optimization of neurons in feed-forward architecture of ANN for the minimization of fitness functions in Equation (21).

Sequential quadratic programming (SQP) is an effective approach for the numerical approximation of linear and non-linear multi-objective optimization problems (NLP) with non-linear constraints. The fundamental concept of sequential quadratic programming is to utilise a quasi-Newton updating method to generate an approximation of the computationally intensive complete Hessian matrix. This causes a sub-problem of quadratic programming to be generated at each iteration; this sub-problem is referred to as a QP sub-problem, and the solution to this sub-problem can be utilised to define the search direction and the next trial solution [49]. Backed by a solid theoretical and computational base, the SQP algorithm has been extensively applied in both commercial and public domains to find solutions for an exceptionally large number of significant practical problems, such as the transient heat conduction problem [50], the non-linear predictive control model [51], life-cycle optimization problems with non-linear state constraints [52], the water wave optimization problem [53] and the design of heating systems in electric heating [54]. Figure 4 illustrates the suggested process for the ANN-TTA-SQP including its granularity.

## 4. Results and Discussion

The details of the implementation, validation and numerical (statistical) analysis of the suggested (ANN-TTA-SQP) paradigm to explore the effects of different parameters (e.g., wet porous parameter, non-dimensional ambient temperature, convection parameter, in-homogeneity index, radiation, and power index) on the thermal distribution of a fin with linear, quadratic and exponential thermal conductivities are discussed in this section. A detailed comparison is presented between the results obtained using the suggested technique (ANN-TTA-SQP) and those obtained using the particle swarm optimization (PSO) algorithm [55,56], the grey wolf optimization (GWO) algorithm [57], the whale optimization algorithm (WOA) [58], the cuckoo search algorithm (CSA) [59], and a data-fitting-based machine learning strategy [60], as shown in Table 1. The accuracy of the results is tested by the values of mean square error. The percentage absolute errors in the solutions of thermal distribution of a porous heat exchanger with different (i.e., linear, quadratic and exponential) heat conductance profiles are shown in Table 2. The values of the percentage absolute errors for different scenarios range between $10^{-2}$ to $10^{-4}$ which validates the efficiency of the solutions. The closed form of the approximate solutions for linear, quadratic and exponential FGM fins for variations in $\theta_{a}$ with $Nr=5,Nc=10,\beta =p=m_{2}=\alpha =1$ are given in Appendix A.

**Figure 5:**
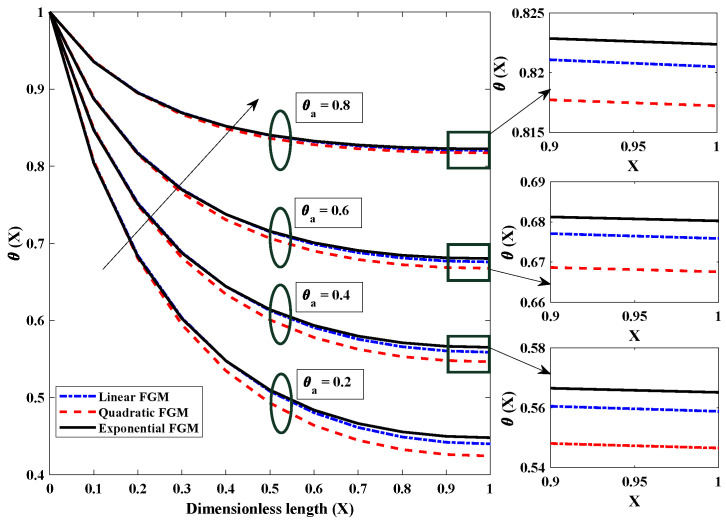
The effect of $\left(\theta_{a}\right)$ surrounding temperature on the heat dispersion profile of the fully wetted longitudinal porous fin with $Nr=5,Nc=10,\beta =p=m_{2}=\alpha =1$.

The influence that the surrounding (ambient) temperature has on the thermal distribution of the FGM heat exchanger with different thermal conductivities is shown in Figure 5. Of note is that the thermal profile continues to decline throughout the axial length of the fin; in addition, a lower thermal profile is observed for lower ambient temperatures. The air temperature surrounding the porous heat exchanger’s surface affects the rate at which heat is transferred away from the fin. The thermal difference between the surroundings and the fin becomes less pronounced as the temperature of the surrounding area increases. According to the rule of cooling established by Newton, there will be a consequent reduction in the cooling effect of the fin. It is clear, based on what can be seen in the figure, that the heat transmission rate is greater in FGM fins with exponential thermal conductivity than in those with linear or quadratic thermal conductance.

**Figure 6:**
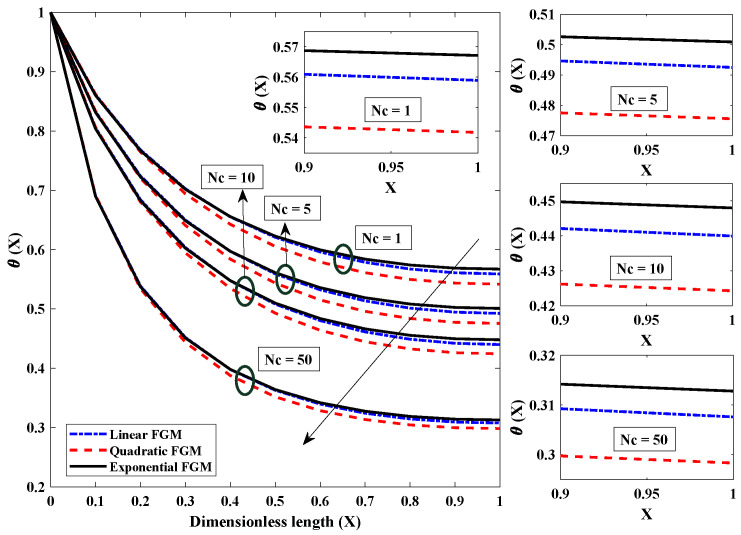
Graphical illustration of influence of convection parameter on thermal profile of the linear, quadratic and exponential FGM fin with $Nr=5,\theta_{a}=0.2,\beta =p=m_{2}=\alpha =1$.

**Figure 7:**
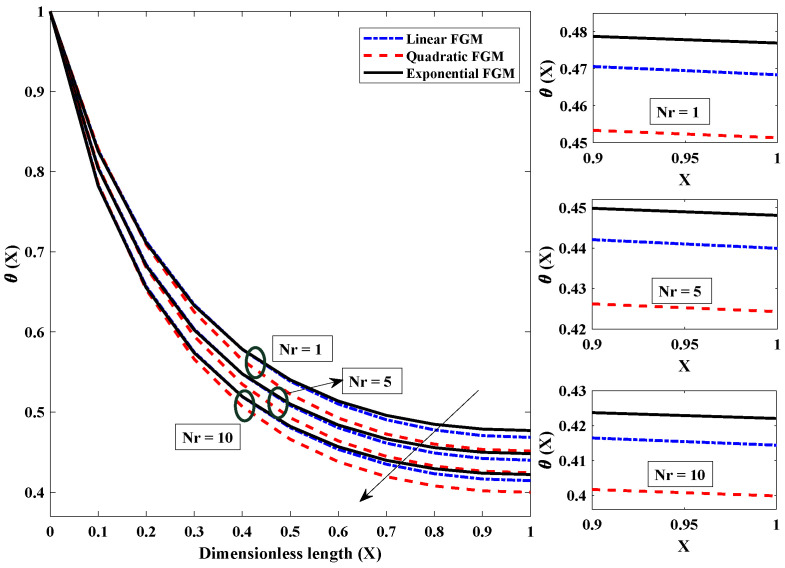
Demonstration of influence of radiation parameter on temperature distribution of FGM fin with $Nc=10,\theta_{a}=0.2,\beta =p=m_{2}=\alpha =1$.

**Figure 8:**
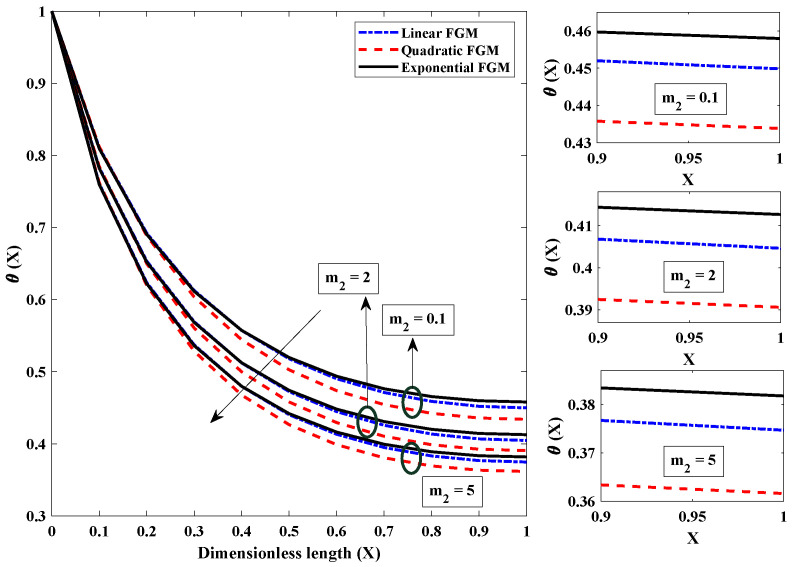
Significance of $\left(m_{2}\right)$, parameter for a moist porous medium on the heat dispersion profile of linear, quadratic and exponential FGM fins with $Nr=5,Nc=10,\beta =p=\alpha =1,\theta_{a}=0.2$.

Figure 6 is plotted to investigate how convection parameter variations affect the fin’s temperature profile. Convection around the FGM fin involves a transfer (loss) of heat/energy to the surroundings. It was observed that when the convective parameter was amplified, i.e., from 1 to 50, there was a rapid decrease in the temperature of the FGM fin. The temperature of the fin dropped as a direct result of an improvement in the convective state, which caused rapid heat transfer from the fin to the surroundings. Because of this, it can be deduced that lower values of the convective parameter are preferable for the thermal properties of the fin in terms of its efficiency. The significance of the radiation parameter on the temperature field of the fully wetted longitudinal fin is seen in Figure 7. The temperature of the heat exchanger’s tip dropped rapidly because heat was lost through the process of radiative heat transfer. In addition, when the values of Nr rose from 1 to 10, the temperature decreased exponentially at a particular axial point. This resulted in a faster cooling of the heat exchanger. Figure 8 shows the effect of the value of the parameter for moist porous media on the thermic profile of the FGM heat exchanger. It is evident that the wet environment surrounding the fin contributes to the absorption of additional heat originating from the top of the fin, which decreases the fin’s temperature profile. As a result, when the wet porous parameter is improved, there is a higher level of heat exchange on the surface of the fin, and the temperature profile is observed to be less severe.

**Figure 9:**
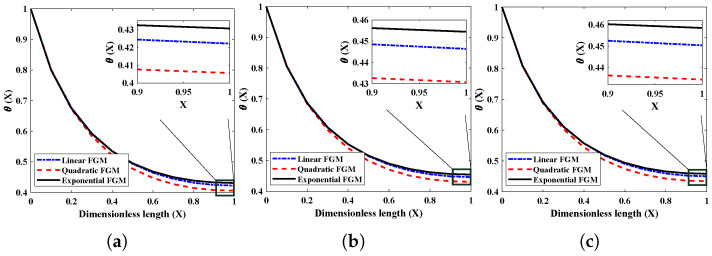
Illustration of variations in power index on temperature distribution of fully wetted longitudinal fin for different thermal conductivities with $Nr=5,Nc=10,\beta =m_{2}=\alpha =1,\theta_{a}=0.2$. (**a**) p=0, (**b**) p=1, (**c**) p=2.

**Figure 10:**
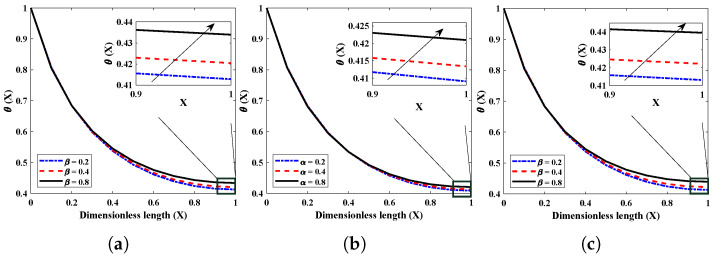
Impact of variations in α and β (inhomogeneity index) on thermal profiles of fully wetted longitudinal fin for different thermal conductivities with $Nr=5,Nc=10,m_{2}=1,\theta_{a}=0.2$. (**a**) p=0, (**b**) p=1, (**c**) p=2.

The relative importance of the convective heat transfer coefficient is indicated by the sign *p* and is referred to as the exponent. Depending on the value of the power index, a different fluid flow regime will occur. The influence of the power index on the temperature performance of a fully wetted FGM fin is illustrated in Figure 9. A closer examination of the data demonstrates that an increase in the value of *p* leads to a rise in temperature all the way down the axial length. This results in a lower rate of heat transfer. As a result, a smaller value for *p* will result in a greater cooling impact. Figure 10 illustrates the impact of variations in α and β (inhomogeneity index) on the dimensionless thermal profile, as well as the rate of heat transfer from the fin. It is clear that a higher value for the non-homogeneous index results in a faster rate of heat transfer. The non-linear condition is also improved by increasing the in-homogeneity index. The quadratic FGM exhibits the lowest temperature out of the three different examples of thermal conductivity, while the exponential FGM fin exhibits the greatest temperature.

## 5. Statistical Analysis

In this section, the results of comparative studies are presented using statistical results from several iterations to determine the accuracy, stability, and convergence of the proposed paradigm. To calculate the solutions for the thermal profile of the fin given in Equations (14)–(16), a developed soft computing paradigm was executed for a hundred individual tests/runs. The errors in the solutions for different variants in ambient temperature with $Nr=5,Nc=10,\beta =p=m_{2}=\alpha =1$ were calculated based on performance functions, such as Theil’s inequality coefficient (TIC), root mean square error (RMSE), mean absolute deviations (MAD), and error in the Nash–Sutcliffe efficiency (ENSE). The mathematical representation of the various performance indicators is as follows:

(31) $$\mathrm{TIC}=\frac{\sqrt{\frac{1}{N}\sum_{k=1}^{N}{\left(\hat{\theta }\left(X_{i}\right)-\theta \left(X_{i}\right)\right)}^{2}}}{\sqrt{\frac{1}{N}\sum_{k=1}^{N}{\left(\hat{\theta }\left(X_{i}\right)\right)}^{2}}+\sqrt{\frac{1}{N}\sum_{k=1}^{N}{\left(\theta \left(X_{i}\right)\right)}^{2}}},$$

(32) $$\mathrm{RMSE}=\sqrt{\frac{1}{N}\sum_{k=1}^{N}{\left(\hat{\theta }\left(X_{i}\right)-\theta \left(X_{i}\right)\right)}^{2}},$$

(33) $$\mathrm{MAD}=\frac{1}{N}\sum_{k=1}^{N}\left(\left|\hat{\theta }\left(X_{i}\right)-\theta \left(X_{i}\right)\right|\right),$$

(34) ENSE=|1−NSE|,

where, NSE is the Nash–Sutcliffe efficiency and is defined as

(35) $$\mathrm{NSE}=1-\left(\frac{\sum_{k=1}^{N}{\left(\hat{\theta }\left(X_{i}\right)-\theta \left(X_{i}\right)\right)}^{2}}{\sum_{k=1}^{N}{\left(\theta \left(X_{i}\right)-\frac{1}{N}\sum_{k=1}^{N}\left(\theta \left(X_{i}\right)\right)\right)}^{2}}\right),$$

where, $\hat{\theta }$ and θ are the approximate and reference solutions, respectively, and *N* denotes the number of grid points.

**Figure 11:**
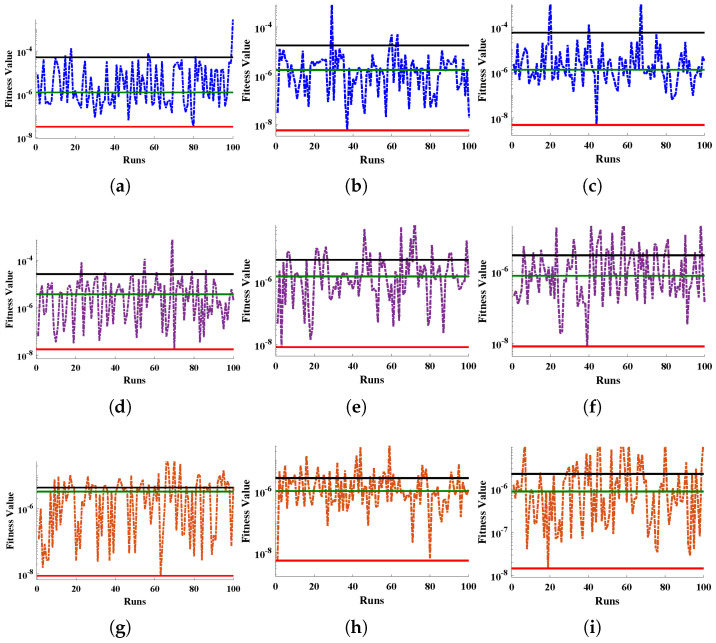
Graphicalillustration of the behaviour of objective function during minimization using the proposed hybrid algorithm for approximate solutions of fully wetted longitudinal fin with (**a**–**c**) linear (**d**–**f**) quadratic and (**g**–**i**) exponential thermal conductivities. Here, red, green and black lines represents the minimum, median and mean values of each case.

**Figure 12:**
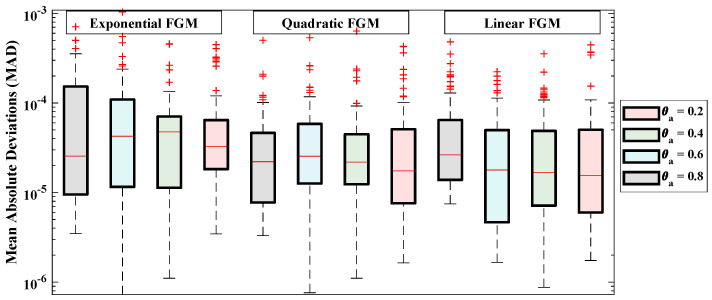
Boxplot analysis of the MAD values obtained during 100 runs of the proposed algorithm. The red lines shows the median value; the upper and lower quartiles represent the maximum and minimum values during the multiple executions.

**Figure 13:**
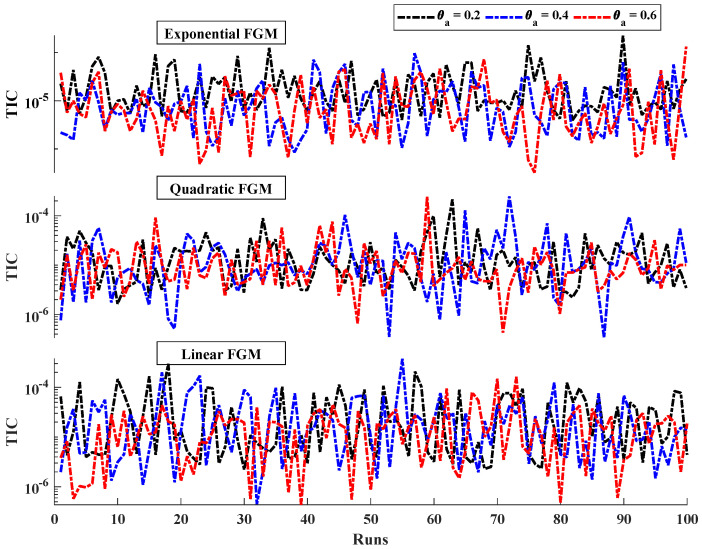
Convergence of TIC values for linear, quadratic and exponential cases of wetted longitudinal porous fin.

**Figure 14:**
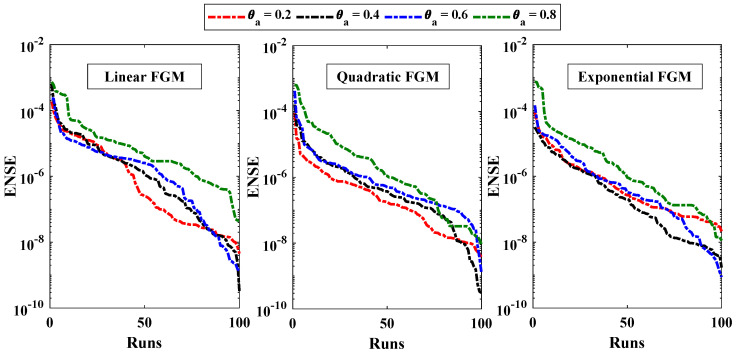
Analysis on ENSE values.

Table 3 demonstrates the statistics of the error functions calculated during the multiple executions. The mean objective values for each case lie around $10^{-6}$ to $10^{-5}$ with standard deviations of $10^{-11}$ to $10^{-10}$. The minimum values of MAD, TIC, RMSE and ENSE lie between $10^{-7}$ to $10^{-6}$, $10^{-8}$ to $10^{-7}$, $10^{-7}$ to $10^{-5}$, and $10^{-10}$ to $10^{-8}$, respectively. Further, the convergence and stability of the results is demonstrated by the results of objective value during 100 runs, as shown in Figure 11. The average distance between the approximated solution and reference solution is shown through Figure 12. The median values for linear, quadratic and exponential FGM lie around $10^{-5}$ to $10^{-4}$ which confirms the correctness of the estimated solutions. The results of TIC and ENSE are plotted in Figure 13 and Figure 14. The values of the performance indicators are close to zero reflecting the accuracy, stability and reliability of the modeled surrogate solutions.

## 6. Conclusions

The thermal behaviour of a porous longitudinal heat exchanger under completely wetted circumstances with linear, quadratic, and exponential thermal conductivities in the presence of convection, conduction, and radiative environments was investigated. Some implications of the results of the numerical experiments undertaken are discussed below.

- A novel unsupervised framework for an intelligent method was designed to construct surrogate solutions for the governing non-linear mathematical model of a fully wetted longitudinal FGM fin. The ANN-TTA-SQP algorithm was implemented to investigate the significance of variations in the dimensionless ambient temperature, parameter for a moist porous medium, convection parameter, in-homogeneity index, radiation parameter, and power index on the temperature distribution of the FGM fin with multiple fluctuations in thermal conductance.
- The approximate solutions obtained were validated by comparing the statistics with state-of-the-art-techniques, including the particle swarm optimization (PSO) algorithm, the cuckoo search algorithm (CSA), the whale optimization algorithm (WOA), the grey wolf optimization (GWO) algorithm and the machine learning algorithm. Minimum values of the mean square errors were observed in the solutions of the proposed technique.
- The thermal distribution in the fin fell when the values of the convective coefficient, radiation coefficient, and parameter for a moist porous medium increased. Increase in the ambient temperature, power index and inhomogeneity parameters caused an increase in the dispersion of the temperature over the heat exchanger.
- Extensive, graphical and statistical analyses were conducted on different error functions, as shown in Figure 11 and Figure 14 and Table 3. The results of these error functions were close to zero, highlighting the approximate solutions’ accuracy and stability.

The results demonstrate the broad applicability, ease of implementation and the ability of the meta-heuristic ANN-TTA-SQP technique to generate optimal solutions for complex engineering problems using an unsupervised approach. The suggested method is highly efficient, but there is a possibility that an increase in the number of layers of ANN might result in an increase in complexity, which would then lead to an increase in the computing cost of the approach. In future, the authors intend to extend the applicability of the proposed algorithm to solve fractional differential equations with ease of implementation. 

## Figures and Tables

**Table 1:** Examination of the differences between the approximated results and mean square errors obtained by the proposed algorithm with PSO, CSA, GWO, and FFNN-BLM algorithms for thermal distribution of fully wetted longitudinal FGM fin with linear thermal conductivity for $\theta_{a}=0.6$, $Nr=5,Nc=10,\beta =p=m_{2}=\alpha =1$.

	Approximate Solution						Mean Square Errors					
X	**PSO**	**CSA**	**WOA**	**GWO**	**FFNN-BLM**	**ANN-TTA-SQP**	**PSO**	**CSA**	**WOA**	**GWO**	**FFNN-BLM**	**ANN-TTA-SQP**
0	0.999711	1.000306	0.999667	0.999504	0.999945	1.000001	$2.275\times 10^{-8}$	$5.081\times 10^{-8}$	$8.432\times 10^{-9}$	$3.743\times 10^{-8}$	$1.407\times 10^{-9}$	$2.148\times 10^{-11}$
0.1	0.887736	0.888135	0.887723	0.887571	0.887901	0.887954	$8.253\times 10^{-7}$	$1.414\times 10^{-7}$	$2.609\times 10^{-7}$	$1.404\times 10^{-6}$	$1.223\times 10^{-7}$	$2.037\times 10^{-10}$
0.2	0.817149	0.817429	0.817140	0.817001	0.817260	0.817297	$4.688\times 10^{-6}$	$9.814\times 10^{-7}$	$6.690\times 10^{-7}$	$7.370\times 10^{-6}$	$8.965\times 10^{-7}$	$1.702\times 10^{-9}$
0.3	0.770009	0.770241	0.770031	0.769846	0.770111	0.770156	$2.351\times 10^{-6}$	$8.038\times 10^{-7}$	$4.663\times 10^{-7}$	$3.568\times 10^{-6}$	$7.725\times 10^{-7}$	$4.539\times 10^{-9}$
0.4	0.737502	0.737702	0.737539	0.737317	0.737594	0.737637	$1.765\times 10^{-6}$	$1.834\times 10^{-7}$	$1.238\times 10^{-7}$	$2.665\times 10^{-6}$	$2.078\times 10^{-7}$	$7.022\times 10^{-10}$
0.5	0.714736	0.714904	0.714770	0.714520	0.714809	0.714842	$6.404\times 10^{-7}$	$2.978\times 10^{-7}$	$2.256\times 10^{-7}$	$1.286\times 10^{-6}$	$3.261\times 10^{-7}$	$1.051\times 10^{-9}$
0.6	0.698784	0.698937	0.698823	0.698519	0.698847	0.698884	$2.715\times 10^{-6}$	$4.156\times 10^{-7}$	$3.335\times 10^{-7}$	$4.165\times 10^{-6}$	$5.494\times 10^{-7}$	$2.095\times 10^{-11}$
0.7	0.687842	0.688008	0.687907	0.687509	0.687915	0.687972	$2.980\times 10^{-7}$	$3.983\times 10^{-10}$	$1.082\times 10^{-10}$	$9.148\times 10^{-8}$	$2.936\times 10^{-9}$	$2.581\times 10^{-10}$
0.8	0.680782	0.680978	0.680884	0.680373	0.680877	0.680957	$1.521\times 10^{-6}$	$5.003\times 10^{-7}$	$4.015\times 10^{-7}$	$4.100\times 10^{-6}$	$5.490\times 10^{-7}$	$4.603\times 10^{-11}$
0.9	0.676882	0.677108	0.677015	0.676395	0.676995	0.677087	$2.711\times 10^{-6}$	$4.119\times 10^{-7}$	$3.176\times 10^{-7}$	$4.277\times 10^{-6}$	$5.757\times 10^{-7}$	$6.330\times 10^{-12}$
1	0.675655	0.675904	0.675807	0.675080	0.675777	0.675874	$1.872\times 10^{-6}$	$6.511\times 10^{-7}$	$6.444\times 10^{-7}$	$6.155\times 10^{-6}$	$6.027\times 10^{-7}$	$1.375\times 10^{-10}$

**Table 2:** Thepercentages of absolute errors in the solutions that were calculated by the ANN-TTA-SQP method for different values of $\theta_{a}$ with $Nr=5,Nc=10,\beta =p=m_{2}=\alpha =1$.

	Linear FGM				Quadratic FGM				Exponential FGM			
X	0.2	0.4	0.6	0.8	0.2	0.4	0.6	0.8	0.2	0.4	0.6	0.8
0.00	0.00005	0.00028	0.00046	0.00077	0.00018	0.00057	0.00013	0.00019	0.00077	0.00021	0.00040	0.00035
0.10	0.00006	0.00118	0.00143	0.00450	0.00186	0.00219	0.00116	0.00064	0.00035	0.00042	0.00056	0.00317
0.20	0.00093	0.00531	0.00413	0.00999	0.00538	0.00309	0.00361	0.00110	0.00020	0.00200	0.00176	0.00963
0.30	0.00242	0.00696	0.00674	0.00665	0.00858	0.00528	0.00315	0.00167	0.00169	0.00436	0.00437	0.00989
0.40	0.00531	0.00040	0.00265	0.00336	0.00184	0.00312	0.00179	0.00118	0.00057	0.00351	0.00475	0.00268
0.50	0.00460	0.00257	0.00324	0.00206	0.00475	0.00372	0.00220	0.00149	0.00320	0.00235	0.00353	0.00651
0.60	0.00243	0.00220	0.00046	0.00516	0.00208	0.00098	0.00277	0.00072	0.00085	0.00272	0.00333	0.00497
0.70	0.00680	0.00163	0.00161	0.00362	0.00192	0.00347	0.00005	0.00017	0.00320	0.00320	0.00484	0.00193
0.80	0.00265	0.00210	0.00068	0.00343	0.00431	0.00159	0.00258	0.00118	0.00302	0.00112	0.00091	0.00535
0.90	0.01247	0.00690	0.00025	0.00864	0.00267	0.00162	0.00190	0.00288	0.00119	0.00409	0.00593	0.00196
1.00	0.00668	0.00535	0.00117	0.00651	0.00460	0.00030	0.00227	0.00382	0.00353	0.00231	0.00432	0.00367

**Table 3:** Statistical analysis of the performance indicators (i.e., fitness value, MAD, TIC, ENSE) for different cases obtained during multiple executions of the designed approach.

FGM		Objective Value		MAD		TIC		RMSE		ENSE	
	$\theta_{a}$	Min.	Avg.	Min.	Avg.	Min.	Avg.	Min.	Avg.	Min.	Avg.
Linear	0.2	$3.15593\times 10^{-8}$	$5.23053\times 10^{-5}$	$3.48355\times 10^{-6}$	$9.05907\times 10^{-5}$	$2.23690\times 10^{-6}$	$3.84823\times 10^{-5}$	$5.69178\times 10^{-6}$	$9.78890\times 10^{-5}$	$4.52603\times 10^{-9}$	$8.41693\times 10^{-6}$
	0.4	$1.73192\times 10^{-8}$	$2.63399\times 10^{-5}$	$7.26234\times 10^{-7}$	$8.60390\times 10^{-5}$	$4.51628\times 10^{-7}$	$3.19952\times 10^{-5}$	$1.31699\times 10^{-6}$	$9.32698\times 10^{-5}$	$3.16279\times 10^{-10}$	$1.52964\times 10^{-5}$
	0.6	$8.69232\times 10^{-9}$	$4.39994\times 10^{-6}$	$1.10382\times 10^{-6}$	$5.75917\times 10^{-5}$	$4.36155\times 10^{-7}$	$1.94033\times 10^{-5}$	$1.43176\times 10^{-6}$	$6.36855\times 10^{-5}$	$1.35244\times 10^{-9}$	$9.66485\times 10^{-6}$
	0.8	$3.48950\times 10^{-8}$	$1.52920\times 10^{-5}$	$3.46206\times 10^{-6}$	$6.72115\times 10^{-5}$	$1.13085\times 10^{-6}$	$2.11378\times 10^{-5}$	$4.22472\times 10^{-6}$	$7.89558\times 10^{-5}$	$4.36061\times 10^{-8}$	$4.49682\times 10^{-5}$
Quadratic	0.2	$5.72905\times 10^{-9}$	$1.77032\times 10^{-5}$	$3.31079\times 10^{-6}$	$3.96153\times 10^{-5}$	$1.64992\times 10^{-6}$	$1.81133\times 10^{-5}$	$4.12421\times 10^{-6}$	$4.52704\times 10^{-5}$	$3.80597\times 10^{-9}$	$1.76396\times 10^{-6}$
	0.4	$8.44298\times 10^{-9}$	$4.85792\times 10^{-6}$	$7.60630\times 10^{-7}$	$4.73085\times 10^{-5}$	$3.37606\times 10^{-7}$	$1.93106\times 10^{-5}$	$9.72378\times 10^{-7}$	$5.56096\times 10^{-5}$	$3.22981\times 10^{-10}$	$4.01069\times 10^{-6}$
	0.6	$5.54459\times 10^{-9}$	$2.70528\times 10^{-6}$	$1.10470\times 10^{-6}$	$4.14064\times 10^{-5}$	$4.27882\times 10^{-7}$	$1.48472\times 10^{-5}$	$1.39391\times 10^{-6}$	$4.83595\times 10^{-5}$	$1.26696\times 10^{-9}$	$7.29760\times 10^{-6}$
	0.8	$3.34359\times 10^{-9}$	$1.10636\times 10^{-5}$	$1.64112\times 10^{-6}$	$4.71864\times 10^{-5}$	$5.47044\times 10^{-7}$	$1.53394\times 10^{-5}$	$2.03774\times 10^{-6}$	$5.71307\times 10^{-5}$	$9.30882\times 10^{-9}$	$2.82836\times 10^{-5}$
Exponential	0.2	$4.92075\times 10^{-9}$	$5.62125\times 10^{-5}$	$7.47090\times 10^{-6}$	$5.71984\times 10^{-5}$	$3.94914\times 10^{-6}$	$2.47741\times 10^{-5}$	$1.00903\times 10^{-5}$	$6.32870\times 10^{-5}$	$2.14259\times 10^{-8}$	$3.41590\times 10^{-6}$
	0.4	$8.20482\times 10^{-9}$	$2.82506\times 10^{-6}$	$1.66266\times 10^{-6}$	$3.57714\times 10^{-5}$	$8.53873\times 10^{-7}$	$1.37292\times 10^{-5}$	$2.49717\times 10^{-6}$	$4.01484\times 10^{-5}$	$1.70630\times 10^{-9}$	$2.04150\times 10^{-6}$
	0.6	$1.47040\times 10^{-8}$	$2.24315\times 10^{-6}$	$8.75080\times 10^{-7}$	$3.81599\times 10^{-5}$	$3.23961\times 10^{-7}$	$1.29419\times 10^{-5}$	$1.06533\times 10^{-6}$	$4.25542\times 10^{-5}$	$8.73426\times 10^{-10}$	$4.80864\times 10^{-6}$
	0.8	$3.24693\times 10^{-8}$	$1.69856\times 10^{-5}$	$1.74177\times 10^{-6}$	$4.71163\times 10^{-5}$	$5.11451\times 10^{-7}$	$1.52643\times 10^{-5}$	$1.91190\times 10^{-6}$	$5.70505\times 10^{-5}$	$1.12716\times 10^{-8}$	$3.52692\times 10^{-5}$

## Data Availability

The data used in this research is available from the corresponding author upon reasonable request.

## Appendix A

The surrogate models of the approximate solution by the proposed algorithm with $\theta_{a}=0.2,Nr=5,Nc=10,\beta =p=m_{2}=\alpha =1$ for linear, quadratic and exponential FGM fins are given as

(A1) $$\begin{array}{cc} \hat{\theta }\left(X\right)= & \frac{5.2319706774108}{1+e^{-(-9.0174471099191X-4.8037378206144)}}\\ \; & +\frac{1.1517807658600}{1+e^{-(10.0153389443112X+2.3861946260666)}}\\ \; & +\frac{5.2603503863984}{1+e^{-(-9.2669382027649X-4.7381310318575)}}\\ \; & +\frac{4.9472485524332}{1+e^{-(-3.3514170802408X-2.7355200886260)}}\\ \; & +\frac{5.2411516708522}{1+e^{-(-9.1013926815623X-4.7827875635947)}}\\ \; & +\frac{5.2575529681820}{1+e^{-(-9.2429522929616X-4.7448830188287)}}\\ \; & +\frac{-4.9400034747211}{1+e^{-(0.3161064753771X-1.6336501804583)}}\\ \; & +\frac{2.8456511470294}{1+e^{-(1.1325396664316X-3.2194699842445)}}\\ \; & +\frac{5.2595364334957}{1+e^{-(-9.2591891766676X-4.7403287007454)}}\\ \; & +\frac{4.9249246883600}{1+e^{-(-2.9605398120784X-3.7004144336356)}}, \end{array}$$

(A2) $$\begin{array}{cc} \hat{\theta }\left(X\right)= & \frac{2.5996762736234}{1+e^{-(0.6936840655374X-3.1464328348186)}}\\ \; & +\frac{5.3991032099844}{1+e^{-(-8.3099946252118X-4.2978574812081)}}\\ \; & +\frac{5.4010296760841}{1+e^{-(-8.4814850547403X-4.2650425389429)}}\\ \; & +\frac{5.0190472445167}{1+e^{-(-3.1038750037864X-2.2568089389813)}}\\ \; & +\frac{5.3971472989380}{1+e^{-(-8.4347989650427X-4.2771012013520)}}\\ \; & +\frac{5.4014731341605}{1+e^{-(-8.4485006001798X-4.2732908438367)}}\\ \; & +\frac{5.3995559736441}{1+e^{-(-8.3903641086452X-4.2859730790917)}}\\ \; & +\frac{3.7570901765922}{1+e^{-(-1.0117997765190X-3.1266718049610)}}\\ \; & +\frac{-5.8729759935637}{1+e^{-(-8.7265158368824X-3.1333538172663)}}\\ \; & +\frac{0.1358079459200}{1+e^{-(5.5948285789310X+9.4505713834768)}}, \end{array}$$

(A3) $$\begin{array}{cc} \hat{\theta }\left(X\right)= & \frac{5.7228769847148}{1+e^{-(-10.1221284100217X-4.5629538618760)}}\\ \; & +\frac{5.7378619596568}{1+e^{-(-10.1747587611975X-4.6041959521860)}}\\ \; & +\frac{2.1067512245631}{1+e^{-(0.4551219479018X-1.9857691604640)}}\\ \; & +\frac{3.0341535540965}{1+e^{-(-4.4354251482154X-2.2908431223331)}}\\ \; & +\frac{5.6788013314162}{1+e^{-(-9.9959724260956X-4.4740986532182)}}\\ \; & +\frac{2.0918110785249}{1+e^{-(-1.8563249944392X-1.4950589293630)}}\\ \; & +\frac{-4.1409229506270}{1+e^{-(-2.3757549644885X-5.7435450648245)}}\\ \; & +\frac{5.6604709547874}{1+e^{-(-9.9538057373586X-4.4497497830332)}}\\ \; & +\frac{-6.0559476407904}{1+e^{-(-10.3200444077758X-3.3416669289871)}}\\ \; & +\frac{5.7261448894420}{1+e^{-(-10.1332450221331X-4.5715300276205)}}. \end{array}$$

The approximate series solutions by the proposed algorithm with $\theta_{a}=0.6$, $Nr=5,Nc=10,\beta =p=m_{2}=\alpha =1$ for linear, quadratic and exponential FGM fins are given as

(A4) $$\begin{array}{cc} \hat{\theta }\left(X\right)= & \frac{5.09123125514838}{1+e^{-(-8.00125135475102X-5.12998804376921)}}\\ \; & +\frac{5.14828586545177}{1+e^{-(-8.61027220800411X-5.01627048086581)}}\\ \; & +\frac{1.46123045178600}{1+e^{-(-3.73819763382428X-2.18468465809794)}}\\ \; & +\frac{4.42747789978089}{1+e^{-(1.47555025377336X-6.09122375819261)}}\\ \; & +\frac{0.11991446542044}{1+e^{-(-1.63963559160162X-0.29203495502782)}}\\ \; & +\frac{4.76283287895868}{1+e^{-(-2.22248850584292X-3.74137659108675)}}\\ \; & +\frac{5.16222820286248}{1+e^{-(-8.73758353998793X-4.98748458228049)}}\\ \; & +\frac{-0.07660777695160}{1+e^{-(-1.67572027828014X-4.96562956333075)}}\\ \; & +\frac{0.60127726611450}{1+e^{-(9.05660017421765X+2.42347804932216)}}\\ \; & +\frac{5.09050622423362}{1+e^{-(-8.00597374664803X-5.12927397595689)}}, \end{array}$$

(A5) $$\begin{array}{cc} \hat{\theta }\left(X\right)= & \frac{5.22517422834494}{1+e^{-(-6.41984648160911X-4.08466173002864)}}\\ \; & +\frac{0.68639691202245}{1+e^{-(-0.71148216272193X-2.60921275409783)}}\\ \; & +\frac{5.22915535992465}{1+e^{-(-6.28304439566354X-3.90235510204152)}}\\ \; & +\frac{0.30332376328545}{1+e^{-(9.30524484225620X+2.11782068420427)}}\\ \; & +\frac{0.64127193088308}{1+e^{-(-2.88158875072922X-2.49313633012203)}}\\ \; & +\frac{3.60184887504373}{1+e^{-(-2.88844246070518X-2.67004217667965)}}\\ \; & +\frac{0.24968104523336}{1+e^{-(6.08237719164033X+1.02914086954798)}}\\ \; & +\frac{1.91262436735248}{1+e^{-(0.76203916763577X-4.47638700854838)}}\\ \; & +\frac{-0.67314751219772}{1+e^{-(-0.58859233819928X-4.22824615678859)}}\\ \; & +\frac{0.81078830289970}{1+e^{-(0.88729465140621X-4.02774045490415)}}, \end{array}$$

(A6) $$\begin{array}{cc} \hat{\theta }\left(X\right)= & \frac{4.58988239718294}{1+e^{-(-16.86864928901090X-9.05074733552389)}}\\ \; & +\frac{5.12197250974732}{1+e^{-(0.56894202922679X-4.32699843562991)}}\\ \; & +\frac{1.86683908487147}{1+e^{-(-3.60402918942503X-2.56293252562351)}}\\ \; & +\frac{3.93853954080051}{1+e^{-(-6.78951822598455X-4.35454589205731)}}\\ \; & +\frac{4.03548111999605}{1+e^{-(-8.01134104175979X-4.4740986532182)}}\\ \; & +\frac{4.73680318573584}{1+e^{-(-16.99433487552000X-8.59731123953630)}}\\ \; & +\frac{4.99138372074869}{1+e^{-(-16.40650186119620X-7.51064988945246)}}\\ \; & +\frac{5.30103774259913}{1+e^{-(-1.92605242359835X-3.37557526725779)}}\\ \; & +\frac{4.90515480883950}{1+e^{-(-16.69462438316830X-7.90735969198721)}}\\ \; & +\frac{0.53322333341375}{1+e^{-(21.26556313830660X+6.70274449755335)}}. \end{array}$$
